# Innovations in Intraocular Lens Power Calculation—A Review

**DOI:** 10.3390/jcm14186585

**Published:** 2025-09-18

**Authors:** Wiktor Stopyra, Andrzej Grzybowski

**Affiliations:** 1 MW-Med Eye Centre, 31-416 Krakow, Poland; 2 Medical Institute, University of Applied Sciences, 34-400 Nowy Targ, Poland; 3 Institute for Research in Ophthalmology, Foundation for Ophthalmology Development, 61-836 Poznan, Poland; ae.grzybowski@gmail.com; 4 Department of Ophthalmology, University of Warmia and Mazury, 10-719 Olsztyn, Poland

**Keywords:** phacoemulsification, intraocular lens power calculation formulas, phacoemulsification, artificial intelligence, root mean square absolute error

## Abstract

**Purpose:** The accurate intraocular lens (IOL) power calculation is essential in phacoemulsification. The latest IOL power calculation formulas, and their new classification and method to assess their accuracy, were described and analyzed. **Design:** Narrative review. **Methods:** The manuscript includes articles on IOL power calculation published between 2019 and 2025. The following key words, such as “phacoemulsification”, “IOL power calculation formula”, “AI-based formulas”, “IOL power selection”, “IOL formulas classification”, IOL prediction” were used to identify papers by searching medical databases (Pubmed/MEDLINE, Google Scholar, Crossref). PRISMA methodology was used to select articles. Finally, 33 peer-reviewed English-language studies with a sample size of at least 120 eyes were included in the analysis. **Results:** Ten IOL power calculation formulas that have been introduced and published over the past 5 years were included in the study. Five of them are artificial intelligence based (Karmona, Hoffer QST, Nallasamy, Zhu-Lu and Zeiss-AI), four are vergence (Emmetropia Verifying Optical, Naeser 2, Voytsekhivskyy Regression Function-Gender and Castrop), and one is ray-tracing (the O formula). In this review, the formulas are introduced and analyzed, with a discussion of selected studies assessing the accuracy of these IOL power calculation methods. **Conclusions:** New IOL power calculation formulas are constantly being developed. They are mostly based on artificial intelligence. New methods are still being sought to assess the accuracy of formulas, and root mean square absolute error is one of them

## 1. Introduction

Sir Nicolas Harold Lloyd Ridley is assumed to be a father of intraocular lens (IOL) implantation, since on 29 November 1949, at St Thomas’ Hospital in London, he successfully implanted a poly(methyl methacrylate) lens; however, postoperatively, the patient had a refractive error of −18.0 D sphere and −6.0 D cylinder [1].

The first wildly recognized IOL power calculation formula was developed by Svyatoslav Nikolaevich Fyodorov in 1967 [2]. The Fyodorov formula, like other early-generation formulas such as Sanders–Retzlaff–Kraff (SRK) [3], was based on axial length (AL) and keratometry (K) measurements. In subsequent years, more advanced formulas were developed, incorporating additional biometric parameters such as anterior chamber depth (ACD), which was introduced in third-generation formulas like Hoffer Q [4], and SRK/T [5]. Over time, numerous other formulas have been proposed [6,7,8,9,10,11,12,13,14], but the debate regarding their accuracy persists, and there is no consensus among cataract surgeons regarding the gold standard for IOL power calculation [15,16,17,18,19,20,21]. The true refractive outcome of an IOL is determined not solely by the precision of pre-surgical biometric measurements such as ACD, AL, and K—where measurement inaccuracies can contribute to 42%, 36%, and 22% of errors, respectively—but also on the effective lens position (ELP) estimation, which is influenced by the choice of the IOL calculation formula [22].

With the development of new formulas came the need to classify them. Over the years, formulas formed generations—from the first to the fourth [5]. However, some authors started to classify formulas by their method of calculating IOL power and the data they use for it [6,23]. In 2017, Koch proposed a classification of formulas based on a logical approach [24]. The rise of artificial intelligence (AI) and its application in IOL power calculation formulas [25,26,27,28,29] led in 2024 to modification of Koch’s classification by Savini, who significantly expanded the segment of AI-based formulas [30].

The accuracy of the IOL power calculation formula is its key aspect [8,10,11,14,16]. In studies evaluating the trueness of IOL power prediction using various formulas, fundamentally, the difference between the predicted outcome preoperatively and the actual outcome achieved postoperatively is the most important [31]. So far, the following parameters, such as refractive prediction error (RPE) including arithmetic mean error, standard deviation (SD), and range, as well as mean absolute error (MAE), median absolute error (MedAE), and percentage of eyes within certain range of PE, i.e., ±0.25 D, ±0.5 D, ±0.75 D and ±1.0 D, have been recommended most often [13,14,16,31]. However, lately, root mean square absolute error (RMSAE) has been readily utilized [17,32].

Although numerous original studies on IOL power calculation have been published, review articles on this topic remain relatively scarce. Moreover, such reviews typically focus on a specific subgroup, for example, IOL power calculation in eyes after corneal refractive surgery [33], with extreme AL [34], or in pediatric patients [35]. In this context, the review by Raimundo and Findl (2025) appears particularly valuable, as it discusses recent advances in IOL power calculation, including newly introduced formulas, comparative analyses between formulas, and emerging trends in the field [36].

This study presents recent innovations in IOL power calculation by introducing and analyzing 10 of the latest formulas as well as discussing selected studies evaluating their accuracy.

## 2. Methods

The methodology follows the Preferred Reporting Items for Systematic reviews and Meta-analyses (PRISMA) guidelines. This review includes papers concerning the latest IOL power calculation formulas introduced and published between 20 August 2019 and 1 March 2025. The following key words, such as “phacoemulsification”, “IOL power calculation formula”, “AI-based formulas”, “IOL power selection”, “IOL formulas classification”, IOL prediction”, were used to identify papers by searching medical databases (Pubmed/MEDLINE, Google Scholar, Crossref). Duplicates were removed during the screening process. During the first phase, the search was limited to abstracts. Eligible studies comprised peer-reviewed papers in English, including original research, reviews, and meta-analyses, while editorials were not considered. After the preliminary hand-search, 33 articles remained for detailed assessment, including studies with the largest sample and the latest formulas. The potential sources of bias in the reviewed studies were systematically evaluated. This involved examining random sequence generation and allocation concealment (selection bias), participants and personnel blinding (performance bias), blinding of outcome evaluators (detection bias), the handling of incomplete data (attrition bias), and the possibility of selective outcome reporting (reporting bias).

## 3. Ethics

This article is based on previously conducted studies and does not contain any new studies with human participants or animals performed by any of the authors.

## 4. Results

**Figure 1:**
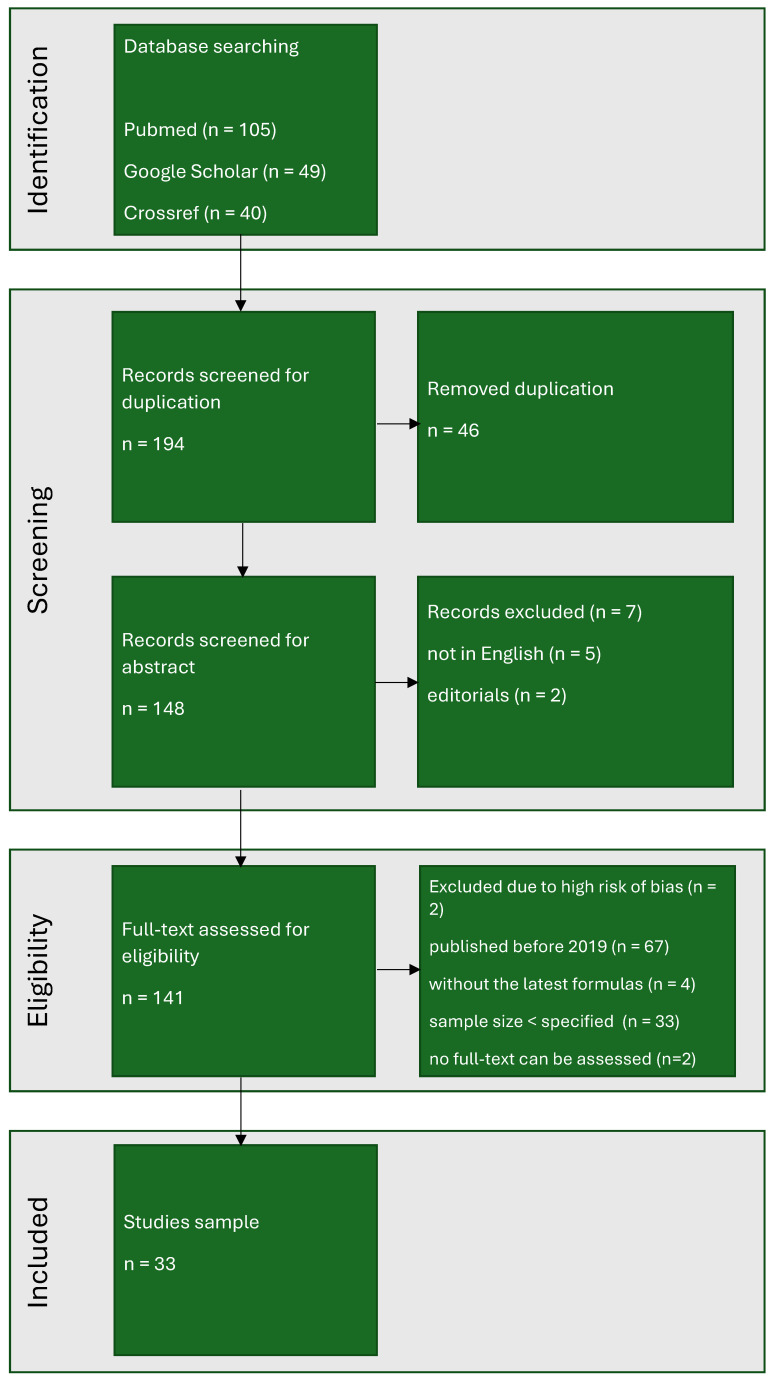
The PRISMA flow chart.

After eliminating duplicates, 148 studies were identified and included in the analysis. Following the preliminary search, 33 publications were chosen for detailed examination. The stepwise procedure of identification, screening, and selection is depicted in the PRISMA flow chart (Figure 1).

## 5. The Latest IOL Power Calculation Formulas

### 5.1. AI-Based Formulas

#### 5.1.1. The Karmona Formula—Solely an AI-Driven Model

The Karmona formula was developed and implemented using the Shiny framework in RStudio (Version 1.1.423; R Foundation, Boston, MA, USA) by David Carmona González (Madrid, Spain, 2018–2021) [25]. This formula represents a novel, artificial intelligence-driven approach to intraocular lens (IOL) power calculation, independent of conventional optical theories such as linear Gaussian optics or ray tracing. The Karmona model is constructed upon two integrated machine learning algorithms, selected from a pool of eleven nonlinear regression models based on their superior predictive performance. These models were subsequently subjected to extensive hyperparameter tuning and optimization processes to enhance their accuracy and generalizability. The final predictive system is an ensemble model that combines outputs from a regularized Bayesian neural network and a Cubist decision tree, thereby leveraging the strengths of both probabilistic and rule-based learning strategies. The Karmona formula utilizes the following input parameters: AL, ACD, WTW distance, and the central radius of the anterior corneal surface. Optionally, it may also incorporate additional biometric variables, including lens thickness (LT), the central radius of the posterior corneal surface, anterior segment depth (defined as the sum of ACD and LT), the ratio of posterior to anterior corneal surface radii, and patient gender. The initial version of the Karmona formula was trained using a dataset comprising 386 eyes, predominantly with AL values ranging from 22.0 mm to 25.0 mm. Similarly to other AI-based systems, the model is designed to undergo continuous learning and retraining, allowing for progressive enhancement of its predictive accuracy over time. By incorporating unique anatomical parameters—particularly anterior segment depth and the posterior-to-anterior corneal curvature ratio—the Karmona formula aims to improve refractive outcomes in eyes with atypical or extreme biometric features. The Karmona formula is available at https://karmona-iol.com (accessed on 10 July 2025).

#### 5.1.2. The Hoffer QST Formula—A Machine Learning–Enhanced Evolution of a Classic Vergence-Based Model

In 2021, Kenneth Hoffer (USA), Giacomo Savini (Italy), and Leonardo Taroni (Italy) introduced the Hoffer QST formula as an advancement of the third-generation, vergence-based Hoffer Q formula through the integration of AI techniques [26]. The primary objective of this enhancement was to improve refractive accuracy, particularly in eyes with long AL. To achieve this goal, a customized AL adjustment was developed using a dedicated dataset comprising over 300 eyes with ALs greater than 25.0 mm. Additionally, the original ACD prediction component of the Hoffer Q formula was replaced with a personalized ACD and a novel T-factor. These parameters were derived using a linear machine learning model trained on an independent dataset of 537 eyes. The training set included biometric inputs such as K, AL, ACD (measured from the corneal epithelium to the crystalline lens), and patient gender. The Hoffer QST formula is publicly accessible via an online platform: https://hofferqst.com (accessed on 10 July 2025).

#### 5.1.3. The Nallasamy Formula—A Two-Layer Ensemble Machine Learning Model

In 2022, Tingyang Li, Joshua Stein, and Nambi Nallasamy introduced a novel intraocular lens (IOL) power calculation formula based on a dataset comprising 5016 patients [28]. Preoperative biometric data were collected at the University of Michigan’s Kellogg Eye Center using Lenstar LS 900 optical biometers (Haag-Streit USA, Mason, OH, USA, EyeSuite software version i9.1.0.0). The authors developed a two-layer ensemble machine learning architecture. In the first layer, a group of level-1 base learners was trained using preoperative biometric measurements and implanted IOL power as input features, with postoperative refraction serving as the prediction target. The second layer consisted of a meta-model that utilized the outputs of the level-1 models as input variables. Accordingly, the number of input features for the level-2 meta-model equaled the number of level-1 learners. The final refractive prediction was generated exclusively by this second-layer meta-model. The Nallasamy formula requires five core biometric inputs: AL, K, ACD, WTW distance, and LT. CCT may be optionally included to further refine prediction accuracy. Nallasamy has been customized for the Alcon SN60WF lens (Alcon, Fort Worth, TX, USA), and additional data will be needed to adjust the method for additional lens models. An online implementation of the Nallasamy formula is available at https://lenscalc.com (accessed on 10 July 2025).

#### 5.1.4. The Zhu-Lu Formula—A Machine Learning–Based Ensemble Model for Highly Myopic Eyes

In June 2023, Yi Lu, Xiangjia Zhu, and colleagues in China introduced the Zhu-Lu formula, specifically developed for intraocular lens (IOL) power calculation in highly myopic eyes. The model was trained and validated on a dataset comprising 1828 eyes, with 1462 allocated to the training set and 366 to the test set [27]. Two distinct sets of input features were constructed to support model training. Set 1 included predicted refractions from both the Haigis and SRK/T formulas, while Set 2 utilized only predictions from the Haigis formula. Each feature set was used to train two supervised machine learning algorithms: eXtreme Gradient Boosting and support vector regression, resulting in four independent sub-models capable of estimating IOL power for highly myopic eyes. To improve predictive robustness and accuracy, the final output was derived by aggregating the predictions from all four sub-models using a weighted averaging technique, yielding a comprehensive ensemble prediction model. The algorithm was implemented in Python 3.7 using the *scikit-learn* library. The Zhu-Lu formula requires six input parameters: AL, K, ACD, LT, WTW distance, and patient gender. The formula is publicly accessible via an online calculator: https://hm-zlf.com (accessed on 10 July 2025).

#### 5.1.5. The Zeiss-AI Formula—A Hybrid Model Integrating AI and Paraxial Ray Tracing

In July 2023, Peter Kenny, Douglas Koch, Warren Hill, and Li Wang published the initial results of the Zeiss AI formula—an advanced IOL power calculation method that integrates AI with paraxial ray tracing principles [29]. Unlike purely data-driven statistical models, the Zeiss AI formula is designed to first learn the underlying physical optics of the human eye. This foundational knowledge is then refined through optimization using thousands of real-world clinical cases for each IOL model, encompassing a wide range of AL, including short, medium, and long eyes. The model requires five essential input parameters: AL, ACD, LT, patient age, and gender. As of now, the Zeiss AI formula is available exclusively through an online calculator restricted to users within the United States.

### 5.2. Vergence Formulas

#### 5.2.1. The Emmetropia Verifying Optical (EVO) Formula—A Thick-Lens IOL Power Model Based on Emmetropization Theory

Developed in 2019 by Tun Kuan Yeo (Singapore), the EVO formula introduces a novel approach to IOL power calculation, grounded in the theory of emmetropization. This model aims to enhance postoperative refractive results by incorporating an individualized “emmetropia factor,” which accounts for each patient’s unique ocular anatomy. Unlike traditional vergence-based formulas, EVO employs advanced algorithmic techniques capable of adapting to a broad spectrum of IOL geometries and powers, thereby enhancing predictive accuracy across diverse anatomical variations. Version 2.0 of the EVO formula prioritizes AL, K, and ACD as primary predictive inputs. Optional parameters, including LT and CCT, may also be incorporated to improve precision. Notably, recent studies have validated the robustness of the EVO formula, demonstrating consistent accuracy even in the absence of ACD measurements. Version 2.0 has shown particular efficacy in eyes with extreme axial lengths—both short and long—and in toric IOL calculations, underscoring its versatility and clinical applicability. Although the use of artificial intelligence in the development of the formula has not been explicitly described, EVO is firmly rooted in contemporary optical theory and supported by extensive empirical validation. The formula is accessible via an online calculator at www.evoiolcalculator.com (accessed on 10 July 2025) [37].

#### 5.2.2. The Naeser Formulas—Thick- and Thin-Lens Models

In 2019, Kristian Næser introduced the Næser 2 formula—a thick-lens vergence-based model for IOL power calculation [11]. Distinct from conventional formulas that estimate the ELP, the Næser 2 formula directly computes the anatomical postoperative ACD. For each IOL power, it calculates individual LT as well as anterior and posterior curvatures of lens. Postoperative optimization was conducted using an independent dataset of 300 eyes, with AL ranging from 21.46 mm to 27.96 mm. This enabled regression-based adjustments tailored to various IOL types, resulting in consistently accurate refractive outcomes across hyperopic, emmetropic, and myopic eyes. The Næser 2 formula is currently available as a Microsoft Excel spreadsheet (Microsoft Corporation, Redmond, WA, USA) via direct communication with the author. Subsequent developments include the Næser III formula, a thick-lens model that assumes a fixed IOL thickness of 0.62 mm, and the Næser IV formula, which applies a thin-lens approximation with a fixed IOL position located 0.31 mm anterior to the postoperative capsular bag [38].

#### 5.2.3. The Voytsekhivskyy Regression Function–Gender (VRF-G) Formula—A Hybrid Optical and Regression-Based Model

The VRF-G formula was introduced in 2020 as an advanced iteration of the original Voytsekhivskyy Regression Function (VRF) model [39]. The VRF-G formula integrates elements of theoretical optics, regression analysis, and ray tracing, resulting in a sophisticated and multifaceted approach to IOL power prediction. The model employs the optical A-constant traditionally used in the SRK/T formula and incorporates eight biometric parameters. Of these, four are mandatory—AL, K, ACD, and patient gender—while four are optional: LT, WTW distance, CCT, and preoperative refraction. Despite its precision and advanced design, the VRF-G formula is not currently available through an online calculator, which limits its accessibility for routine clinical use. It is distributed exclusively via the standalone VRF Suite software (Version 1.7), which remains under active development.

#### 5.2.4. The Castrop Formula—A Paraxial Vergence-Based Model Incorporating Detailed Ocular Geometry

In 2021, Achim Langenbucher and Peter Hoffmann (Germany) introduced the Castrop formula—a paraxial, vergence-based IOL power calculation model derived from a pseudophakic model eye [40]. This formula uniquely incorporates multiple refractive interfaces, including the spectacle plane, the cornea (modeled via anterior and posterior surface curvatures and CCT), and the IOL itself. The Castrop formula employs three constants:

- C, analogous to the Olsen formula’s constant;
- H, which compensates for systematic shifts in the IOL plane depending on the IOL’s optic and haptic design; and
- R, a refraction offset constant that corrects for systematic prediction errors in postoperative refraction.

The required biometric input parameters include AL, CCT, ACD, LT, the mean anterior corneal radius of curvature (Rmean), and the posterior corneal surface radius. The Castrop formula is publicly accessible via the IOLCon online platform at https://iolcon.org (accessed on 10 July 2025).

### 5.3. Ray-Tracing Formulas

#### The O Formula—A Novel OCT-Based Approach

In 2022, So Goto and al. (Japan) introduced the O formula—an innovative IOL power calculation method that incorporates four key conceptual advancements [41]. First, AL is redefined as the distance from the anterior corneal surface to the photoreceptor layer, measured using a segmented approach and adjusted for cataract grade. Second, the formula predicts IOL depth—defined as the distance from the corneal endothelium to the anterior IOL surface—based on anterior segment parameters obtained via optical coherence tomography (OCT). Third, the model accounts for the actual physical shape and refractive index of the implanted IOL, tailoring calculations to the specific optical properties of each lens power. Fourth, optical corneal power is computed using both anterior and posterior corneal curvatures and central corneal thickness, applying Snell’s law to improve refractive accuracy. The O formula relies on preoperative biometric data acquired through swept-source OCT (SS-OCT) and SS-OCT–based biometers, enabling highly detailed assessment of anterior segment anatomy and enhancing the precision of IOL power prediction.

## 6. Discussion

Precise IOL power calculation is essential in phacoemulsification, as patients increasingly expect excellent visual outcomes after cataract surgery. The continuous development of new formulas, especially those AI-based, led to the need for a revised classification. In 2024, Giacomo Savini et al. proposed a modification of a current classification by Koch [30], using a logical approach. In their framework, formulas are divided into three main groups: vergence-based, AI-based, and ray-tracing-based. The AI-based group is further divided into four subcategories: solely AI, and three hybrid types in which AI refines either thin-lens vergence, thick-lens vergence, or paraxial ray-tracing formulas (Table 1). This updated classification highlights the growing role of AI in IOL power calculation.

The prediction error (PE), defined as the difference between the actual value and the predicted outcome, is a crucial metric for assessing the accuracy of IOL power calculation formulas. The standard deviation (SD) is the most commonly used measure of how data points deviate from the mean. However, this approach assumes that the mean PE is zero, which may not hold true in certain datasets, such as those involving extreme cases (e.g., short eyes, long eyes, or eyes that have undergone corneal refractive surgery). In 2023, Jack Holladay et al. proposed that in such cases, the RMSAE should be used as a more appropriate measure for evaluating formula performance [42]. The equation for RMSAE is as follows:

RMSAE of PE = √Sum(PE)^2^/n

where n is number of samples.

Alternatively, mean absolute error (MAE) can also be used, according to the following equation:

MAE of PE = Sum|PE|/n

Accurate IOL power calculation is critical for achieving optimal visual outcomes in phacoemulsification, particularly as patient expectations continue to rise in the era of refractive cataract surgery [43]. Consequently, the pursuit of increasingly precise IOL calculation formulas remains a central focus in ophthalmology, with AI-based approaches gaining prominence in recent years. Despite their innovative potential, current machine learning–based IOL power calculation methods exhibit several important limitations. First, many studies compare the performance of new formulas primarily against older-generation models, rather than benchmarking them against the most accurate contemporary formulas. Second, some models fail to demonstrate statistically significant improvements in predictive accuracy when compared with current gold-standard methods. Third, the use of relatively small training datasets raises concerns regarding the robustness, reproducibility, and generalizability of these AI-driven algorithms [28].

In 2022, Li et al. presented the Nallasamy formula, a novel machine learning-based IOL power calculation formula developed based on a dataset of 6893 eyes, which achieved significantly better prediction accuracy than five traditional IOL power formulas including Barrett Universal II [28]. This formula obtained a very high Formula Performance Index (FPI) of 0.447. Hoffer et al. recommend calculating FPI as another tool for assessing the accuracy of formulas as follows [44]:

FPI = 1/(SD + MedAE + 10 × abs(m) + 10 × (n10) − 1)

where:

m—slope of the correlation between the arithmetic error and AL;
n—the percentage of eyes with an absolute error within 0.5 D.

Additionally, Nallasamy achieved the lowest MAE and MedAE in all AL subgroups such as short, medium, and long eyes. However, their study has several limitations, such as no validation on a dataset from a different medical institution and no comparison with the performance of other AI-based formulas, i.e., Hill-RBF 3.0, Kane, Pearl-DGS or Hoffer QST.

Another study on a large dataset (1828 eyes) was conducted by Guo et al. in 2023 [27]. They tested six formulas including Zhu-Lu, Barrett Universal II, Kane, Pearl-DGS, EVO 2.0, and Hill-RBF 3.0. Zhu-Lu obtained the highest percentage of eyes with PE within ±0.5 D (80.61%), followed by Hill-RBF 3.0 (72.85%) and Barrett Universal II (62.33%). In addition, Zhu-Lu achieved the lowest MAE (0.34), MedAE (0.26) and SD (0.46); however, its PE was slightly hyperopic (0.005). In the AL subgroup analysis, the PE of the Zhu-Lu stayed stably close to zero in all subgroups, i.e., 26.0–28.0 mm, 28.0–30.0 mm, and ≥30.0 mm. The study was limited to highly myopic eyes so the outcomes do not apply to short and medium eyes, which is a significant limitation of the study.

Taroni et al. carried out an interesting study comprising 1259 eyes in two subgroups—Caucasian and Asian [26]. They examined six formulas such as Hoffer QST, EVO 2.0, Barrett Universal II, Kane, Hill-RBF 3.0, and Hoffer Q. In Caucasian eyes, EVO 2.0 yielded the highest percentage of eyes with PE within ±0.5 D (76.87%) ahead of Hoffer QST (76.29%), and Kane (76.29%). In Asian eyes, the outcomes were, respectively, Hill-RBF 3.0 (88.28%), Hoffer QST (87.21%), and Kane (86.68%). The study includes all AL subgroups, i.e., short, medium, and long eyes, which is its great advantage. However, the retrospective nature and lack of LT measurement is a main limitation.

Recently, Redden et al. published a study based on 886 eyes, including five formulas (Castrop, SRK/T, Haigis, Hoffer Q, and Holladay 1) and three regression-based models, such as classical linear models, regression splines, and random forest regression [45]. Among IOL power calculation formulas, Castrop obtained the lowest RMSAE (0.359) and MAE (0.284); however, it was outperformed by a model where the effects of some covariates (AL, CCT) were modeled as nonlinear via regression splines. The study is promising; however, its retrospective, monocentric design is a limitation. Additionally, excluding Barrett Universal II, EVO 2.0, Hill-RBF, Kane, Pearl-DGS, and Hoffer QST significantly reduces its clinical value.

Thirteen formulas (among others, Kane, EVO 2.0, VRF-G, Barrett Universal II, Naeser 2, Hill-RBF 2.0, and Pearl-DGS) were tested in Hipólito-Fernandes’ study [39]. VRF-G achieved the highest percentage of patients with PE within ±0.5 D (79.5%), followed by Kane (79.3%) and EVO 2.0 (78.5%). A similar order was when considering MedAE (0.273, 0.274, 0.282, respectively). In terms of MAE, 3ane yielded the lowest outcome (0.324), EVO 2.0 the second lowest (0.329) and VRF-G the third lowest (0.332). Contrary to recommendation by Simpson and Charman, the value of postoperative refraction was measured at 4 m [46], which is a significant limitation of the study. In addition, data from various surgeons may introduce bias. However, there are two philosophies regarding the most appropriate method to evaluate the results of IOL power formulas used in clinical practice [47]. Different surgeons ensure generalization of the outcomes.

**Figure 2:**
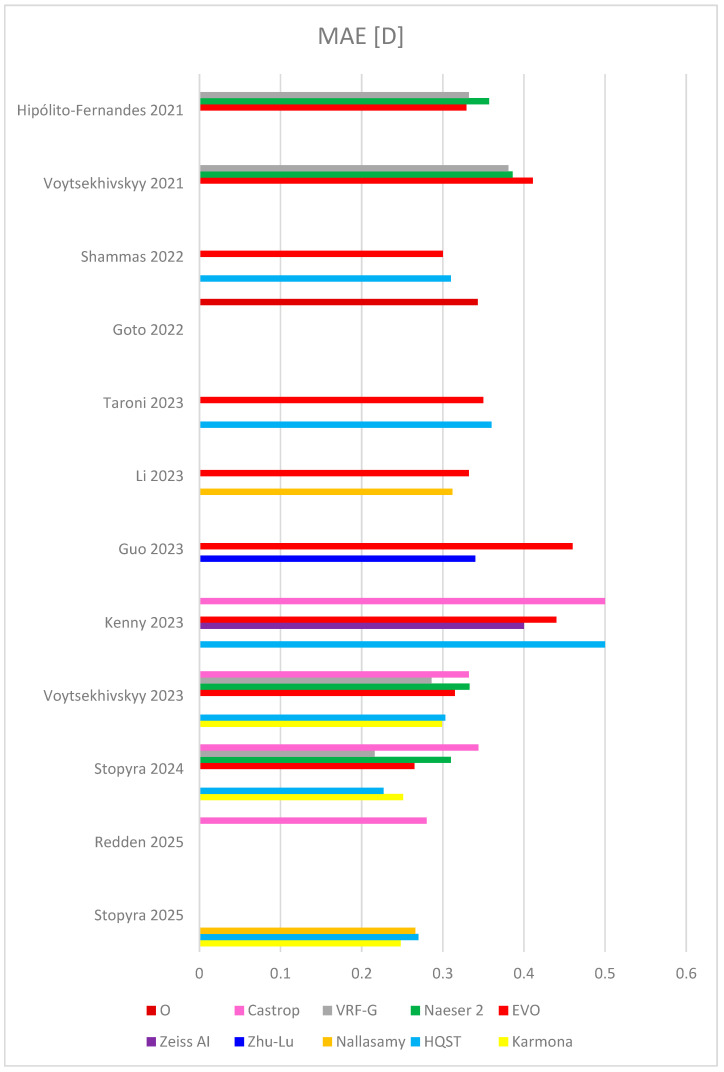
Outcomes of mean absolute error (MAE) in several last studies [26,27,28,29,39,41,45,48,49,50,51,52]. HQST = Hoffer QST.

**Figure 3:**
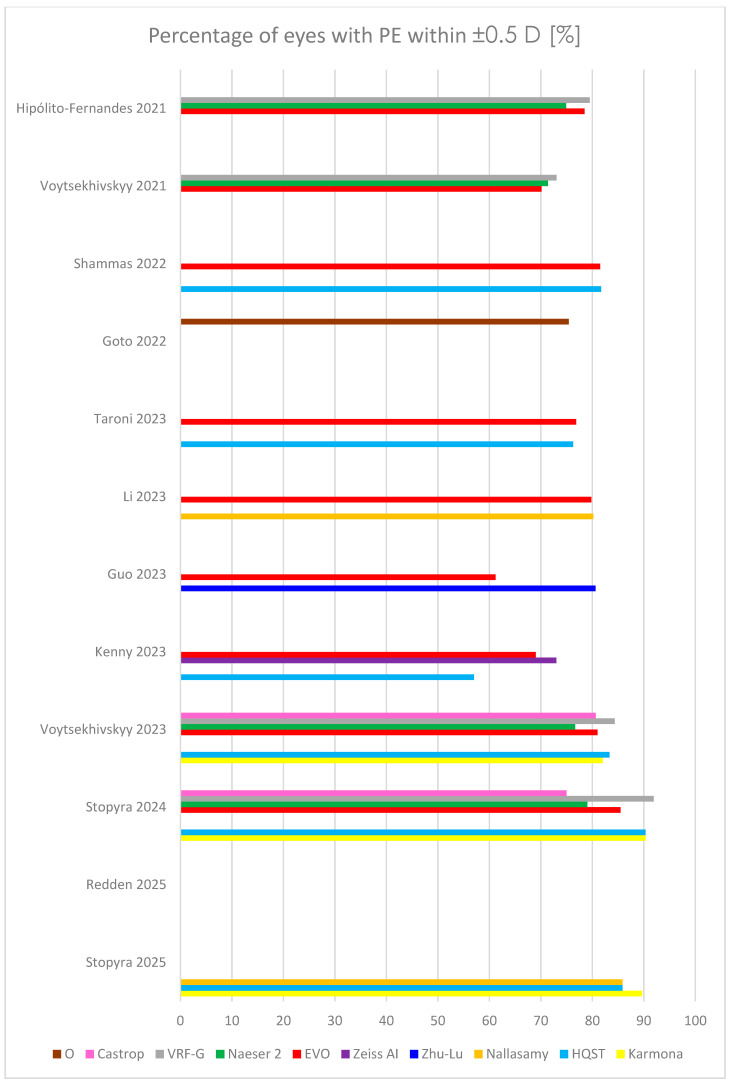
Outcomes of percentage of eyes with prediction error (PE) within ±0.5 D in several last studies [26,27,28,29,39,41,45,48,49,50,51,52]. HQST = Hoffer QST.

In 2022, Shammas published a very robust study, comprising 595 eyes and comparing 17 formulas (among others, Hill-RBF, Kane, Pearl-DGS, Hoffer QST, Barrett Universal II, EVO) [48]. The highest percentage of patients with PE within ±0.5 D was yielded by Pearl-DGS (81.7%), ahead of Hill-RBF (81.5%) and EVO (81.5%). Pearl-DGS and K6 obtained the lowest MedAE and MAE (0.29 and 0.26, respectively). Although the study was carried out on the eyes throughout entire AL, the small sample size of short and long eyes is a limitation of the study. Detailed outcomes of selected newer formulas, including MAE, MedAE, RMSAE, and percentage of eyes with PE within ±0.5 D, from recent studies comprising the largest sample sizes are summarized in Table 2, and Figure 2 and Figure 3.

This study has several limitations. First, the method of achieving the biometric data using biometers based on group refractive index or sum-of-segments technology was not considered. It was proved that group refractive biometers could be affected by lens opacity, and they generally overestimate AL in long eyes and underestimate AL in short eyes [53]. Second, preliminary sample size calculation was not carried out. Third, no meta-analysis was performed. However, it was due to the substantial variability across the analyzed studies (the use of different instruments for biometric data collection, implementation of various IOL models, different surgeons with various skills, a disparate range of biometric parameters such AL and ACD, varied equipment used to achieve postoperative refraction, etc.).

## 7. Conclusions

Accurate IOL power calculation is a very important aspect of cataract surgery [54]. However, according to the European Registry of Quality Outcomes in Cataract and Refractive Surgery, the percentage of prediction error within ±0.5 D after cataract surgery is only 73.7% [55]. This percentage is even lower in special cases, such as in eyes after corneal refractive surgery, vitrectomy, with keratoconus, and with extremely long or short AL, as well as in pediatric eyes. This is the reason for the development of new IOL power calculation formulas. In the last 5 years, at least 10 new formulas have been launched [11,25,26,27,28,29,37,39,40,41]. Mostly, they are based on AI [25,26,27,28,29]. Such a large number of new formulas made it necessary to classify them anew. In 2024, Savini et al. published a new classification that organizes recent formulas [30]. This classification emphasizes AI formulas. Different methods can be used to evaluate the accuracy of IOL power calculation formulas. SD is applied most frequently when the PE is zero. Recently, Holladay proposed RMSAE, for when PE cannot be adjusted to zero [42]. However, despite the many formulas and tools for assessing their accuracy, there is still no consensus among cataract surgeons on which formula to choose for calculating IOL power, so each patient should be treated individually.

## Figures and Tables

**Table 1:** Intraocular lens power calculation formulas.

Group	Subgroup	Formula
**Vergence**	**Thin lens**	Holladay 1
		Hoffer Q
		SRK/T
		Haigis
		Holladay 2
		Castrop
		Cooke K6
		Panacea
		T2
		VRF
		VRF-G
	**Thick lens**	EVO 2.0
		Naeser 2
**Artificial intelligence (AI)**	**Only AI**	Hill-RBF 2.0
		Karmona
		Hill-RBF 3.0
		Nallasamy
	**AI + thin lens**	Ladas SF AI
		3C Calculator
		Kane
		Hoffer QST
		Zhu-Lu
	**AI + thick lens**	Pearl-DGS
	**AI + paraxial**	Zeiss AI
**Ray tracing**	**Paraxial**	Olsen
		Barrett Universal II
		O
	**Exact**	Okulix

**Table 2:** Outcomes of mean absolute error (MAE), median absolute error (MedAE), percentage of eyes with prediction error (PE) within ±0.5 D, and root mean square absolute error (RMSAE) in several recent studies.

Study		Karmona	HQST	Nallasamy	Zhu-Lu	Zeiss AI	EVO	Naeser 2	VRF-G	Castrop	O
Hipólito-Fernandes 2021 (828 eyes) [39]	MAE						0.329	0.357	0.332		
	MedAE										
	±0.5 D						78.5	74.9	79.5		
	RMSAE										
Voytsekhivskyy 2021 (241 eyes) [49]	MAE						0.411	0.386	0.381		
	MedAE						0.325	0.277	0.276		
	±0.5 D						70.12	71.37	73.03		
	RMSAE										
Shammas 2022 (595 eyes) [48]	MAE		0.31				0.30				
	MedAE		0.28				0.27				
	±0.5 D		81.7				81.5				
	RMSAE										
Goto 2022 (423 eyes) [41]	MAE										0.343
	MedAE										0.290
	±0.5 D										75.4
	RMSAE										
Taroni 2023 (1259 eyes) [26]	MAE		0.36				0.35				
	MedAE		0.29				0.30				
	±0.5 D		76.29				76.87				
	RMSAE		0.47				0.46				
Li 2023 (6893 eyes) [28]	MAE			0.312			0.322				
	MedAE			0.242			0.251				
	±0.5 D			80.2			79.8				
	RMSAE										
Guo 2023 (1828 eyes) [27]	MAE				0.34		0.46				
	MedAE				0.26		0.40				
	±0.5 D				80.61		61.22				
	RMSAE										
Kenny 2023 (278 eyes) [29]	MAE		0.50			0.40	0.44			0.50	
	MedAE										
	±0.5 D		57.0				69.0				
	RMSAE		0.56			0.55	0.60			0.66	
Voytsekhivskyy 2023 (300 eyes) [50]	MAE	0.299	0.303				0.315	0.333	0.286	0.334	
	MedAE	0.231	0.227				0.244	0.255	0.209	0.250	
	±0.5 D	82.00	83.33				81.00	76.67	84.33	80.67	
	RMSAE										
Stopyra 2024 (124 eyes) [51]	MAE	0.251	0.227				0.265	0.310	0.216	0.344	
	MedAE	0.229	0.169				0.206	0.228	0.169	0.261	
	±0.5 D	90.32	90.32				85.48	79.03	91.94	75.00	
	RMSAE	0.313	0.298				0.341	0.400	0.286	0.442	
Redden 2025 (886 eyes) [45]	MAE									0.280	
	MedAE										
	±0.5 D										
	RMSAE									0.355	
Stopyra 2025 (184 eyes) [52]	MAE	0.248	0.270	0.266							
	MedAE	0.200	0.220	0.222							
	±0.5 D	89.67	85.87	85.87							
	RMSAE										

HQST = Hoffer QST.

## Data Availability

No new data were created or analyzed in this study. Data sharing is not applicable to this article.

## References

[1] Leffler C.T., Spalton D., Schwartz S.G., Grzybowski A., Maloney R.K. (2025). Sir Harold Ridley (1906–2001) and His Cure for Aphakia: New Historical Insights Into the Invention of the Intraocular Lens. Am. J. Ophthalmol..

[2] Fedorov S.N., Kolinko A.I. (1967). A method of calculating the optical power of the intraocular lens. Vestn. Oftalmol..

[3] Stopyra W. (2022). Effectiveness, Sensitivity and Specificity of Intraocular Lens Power Calculation Formulas for Short Eyes. Turk. J. Ophthalmol..

[4] Hoffer K.J. (1993). The Hoffer Q formula: A comparison of theoretic and regression formulas. J. Cataract Refract. Surg..

[5] Retzlaff J.A., Sanders D.R., Kraff M.C. (1990). Development of the SRK/T intraocular lens implant power calculation formula. J. Cataract Refract. Surg..

[6] Chung J., Bu J.J., Afshari N.A. (2022). Advancements in intraocular lens power calculation formulas. Curr. Opin. Ophthalmol..

[7] Haigis W., Lege B., Miller N., Schneider B. (2000). Comparison of immersion ultrasound biometry and partial coherence interferometry for intraocular lens calculation according to Haigis. Graefes Arch. Clin. Exp. Ophthalmol..

[8] Sheard R.M., Smith G.T., Cooke D.L. (2010). Improving the prediction accuracy of the SRK/T formula: The T2 formula. J. Cataract Refract. Surg..

[9] Hoffer K.J., Savini G. (2017). IOL Power Calculation in Short and Long Eyes. Asia Pac. J. Ophthalmol..

[10] Ladas J.G., Siddiqui A.A., Devgan U., Jun A. (2015). A 3-D “Super Surface” Combining Intraocular Lens Formulas to Generate a “Super Formula” and Maximize Accuracy. JAMA Ophthalmol..

[11] Næser K., Savini G. (2019). Accuracy of thick-lens intraocular lens power calculation based on cutting-card or calculated data for lens architecture. J. Cataract Refract. Surg..

[12] Debellemanière G., Dubois M., Gauvin M., Wallerstein A., Brenner L., Rampat R., Saad A., Gatinel D. (2021). The PEARL-DGS Formula: The Development of an Open-source Machine Learning-based Thick IOL Calculation Formula. Am. J. Ophthalmol..

[13] Voytsekhivskyy O.V. (2018). Development and clinical accuracy of a new intraocular lens power formula (VRF) compared to other formulas. Am. J. Ophthalmol..

[14] Kane J.X., Van Heerden A., Atik A., Petsoglou C. (2017). Accuracy of 3 new method for intraocular lens power selection. J. Cataract. Refract. Surg..

[15] Stopyra W. (2022). Analysis of accuracy of twelve intraocular lens power calculation formulas for eyes with axial myopia. Taiwan J. Ophthalmol..

[16] Darcy K., Gunn D., Tavassoli S., Sparrow J., Kane J.X. (2020). Assessment of the accuracy of new and updated intraocular lens power calculation formulas in 10930 eyes from the UK National Health Service. J. Cataract. Refract. Surg..

[17] Stopyra W., Voytsekhivskyy O., Grzybowski A. (2025). Accuracy of 7 artificial intelligence based intraocular lens power calculation formulas in extremely long Caucasian eyes. Am. J. Ophthalmol..

[18] Ma Y., Xiong R., Liu Z., Young C.A., Wu Y., Zheng D., Zhang X., Jin G. (2024). Network Meta-analysis of Intraocular Lens Power Calculation Formula Accuracy in 1016 Eyes With Long Axial Length. Am. J. Ophthalmol..

[19] Hong Y., Sun Y., Xiao B., Ainiwear M., Ji Y. (2023). A Bayesian network meta-analysis on comparisons of intraocular lens power calculation methods for paediatric cataract eyes. Eye.

[20] Stopyra W., Grzybowski A. (2024). Intraocular Lens Power Calculation Formulas in Children—A Systematic Review. J. Clin. Med..

[21] Melles R.B., Holladay J.T., Chang W.J. (2018). Accuracy of Intraocular Lens calculation Formulas. Ophthalmology.

[22] Kane J.X., Chang D.F. (2021). Intraocular Lens Power Formulas, Biometry, and Intraoperative Aberrometry. A Review. Ophthalmology.

[23] Olsen T. (2007). Calculation of intraocular lens power: A review. Acta Ophthalmol. Scand..

[24] Koch D.D., Hill W., Abulafia A., Wang L. (2017). Pursuing perfection in intraocular lens calculations: I. Logical approach for classifying IOL calculation formulas. J. Cataract Refract. Surg..

[25] Carmona-González D., Palomino-Bautista C. (2021). Accuracy of a new intraocular lens power calculation method based on artificial intelligence. Eye.

[26] Taroni L., Hoffer K.J., Pellegrini M., Lupardi E., Savini G. (2023). Comparison of the New Hoffer QST with 4 Modern Accurate Formulas. J. Cataract Refract. Surg..

[27] Guo D., He W., Wei L., Song Y., Qi J., Yao Y., Chen X., Huang J., Lu Y., Zhu X. (2023). The Zhu-Lu formula: A machine learning-based intraocular lens power calculation formula for highly myopic eyes. Eye Vis..

[28] Li T., Stein J., Nallasamy N. (2023). Evaluation of the Nallasamy formula: A stacking ensemble machine learning method for refraction prediction in cataract surgery. Br. J. Ophthalmol..

[29] Kenny P.I., Kozhaya K., Truong P., Weikert M.P., Hill W.E., Koch D.D. (2023). Efficacy of segmented axial length and artificial intelligence approaches to intraocular lens power calculation in short eyes. J. Cataract Refract. Surg..

[30] Savini G., Hoffer K.J., Kohnen T. (2024). IOL power formulas classifications. J. Cataract Refract. Surg..

[31] Wang L., Koch D.D., Hill W., Abulafia A. (2017). Pursuing perfection in intraocular lens calculations: III. Criteria for analyzing outcomes. J. Cataract Refract. Surg..

[32] Holladay J.T., Wilcox R.R., Koch D.D., Wang L. (2021). Review and recommendations for univariate statistical analysis of spherical equivalent prediction error for IOL power calculations. J. Cataract Refract. Surg..

[33] Moshirfar M., Sperry R.A., Altaf A.W., Stoakes I.M., Hoopes P.C. (2024). Predictability of Existing IOL Formulas After Cataract Surgery in Patients with a Previous History of Radial Keratotomy: A Retrospective Cohort Study and Literature Review. Ophthalmol. Ther..

[34] Moore J.E., McNeely R.N., Moutari S. (2025). Cataract Surgery in the Small Adult Eye: A Review. Clin. Exp. Ophthalmol..

[35] Wójcik-Niklewska B., Nocoń-Bratek M., Szala K. (2025). Intraocular lens power calculation in pediatric cataract surgery: A narrative review. Medicine.

[36] Raimundo M., Findl O. (2025). Update on intraocular lens formulas. Curr. Opin. Ophthalmol..

[37] Savini G., Taroni L., Hoffer K.J. (2020). Recent developments in intraocular lens power calculation methods-update 2020. Ann. Transl. Med..

[38] Naeser K., Nielsen R. (2025). Accuracy of thick and thin intraocular lens power formulas using paraxial vergence calculation. J. Cataract Refract. Surg..

[39] Hipólito-Fernandes D., Luís M.E., Gil P., Maduro V., Feijão J., Yeo T.K., Voytsekhivskyy O., Alves N. (2020). VRF-G, a New Intraocular Lens Power Calculation Formula: A 13-Formulas Comparison Study. Clin. Ophthalmol..

[40] Langenbucher A., Szentmáry N., Cayless A., Weisensee J., Fabian E., Wendelstein J., Hoffmann P. (2021). Considerations on the Castrop formula for calculation of intraocular lens power. PLoS ONE.

[41] Goto S., Maeda N., Ohnuma K., Lawu T., Kawasaki R., Koh S., Nishida K., Noda T. (2022). Preliminary demonstration of a novel intraocular lens power calculation:the O formula. J. Cataract Refract. Surg..

[42] Holladay J.T., Wilcox R.R., Koch D.D., Wang L. (2023). Statistics of prediction error for dependent and independent datasets. J. Cataract Refract. Surg..

[43] Gökce S.E., Zeiter J.H., Weikert M.P., Koch D.D., Hill W., Wang L. (2017). Intraocular lens power calculations in short eyes using 7 formulas. J. Cataract Refract. Surg..

[44] Hoffer K.J., Savini G. (2021). Update on Intraocular Lens Power Calculation Study Protocols: The Better Way to Design and Report Clinical Trials. Ophthalmology.

[45] Redden L.D., Graubauer B., Hoffmann P., Langenbucher A., Riaz K.M., Gatinel D., Wagner H., Wendelstein J.A. (2025). Intraocular Lens Power calculation–Comparing Big Data Approaches to Established Formulas. Am. J. Ophthalmol..

[46] Simpson M.J., Charman W.N. (2014). The effect of testing distance on intraocular lens power calculation. J. Refract. Surg..

[47] Stopyra W., Voytsekhivskyy O., Grzybowski A. (2025). Prediction of Seven Artificial Intelligence-Based Intraocular Lens Power Calculation Formulas in Medium-Long Caucasian Eyes. Life.

[48] Shammas H.J., Taroni L., Pellegrini M., Shammas M.C., Jivrajka R.V. (2022). Accuracy of never IOL power formulas in short and long eyes using sum-of-segment biometry. J. Cataract Refract. Surg..

[49] Voytsekhivskyy O.V., Hoffer K.J., Savini G., Tutchenko L.P., Hipólito-Fernandes D. (2021). Clinical Accuracy of 18 IOL Power Formulas in 241 Short Eyes. Curr. Eye Res..

[50] Voytsekhivskyy O.V., Hoffer K.J., Tutchenko L., Cooke D.L., Savini G. (2023). Accuracy of 24 IOL Power Calculation Methods. J. Refract. Surg..

[51] Stopyra W., Voytsekhivskyy O., Grzybowski A. (2024). Accuracy of 20 Intraocular Lens Power Calculation Formulas in Medium-Long Eyes. Ophthalmol. Ther..

[52] Stopyra W., Voytsekhivskyy O., Grzybowski A. (2025). Accuracy of 7 artificial intelligence-base intraocular lens power calculation formulas in medium-long eyes: 2-center study. Can. J. Ophthalmol..

[53] De Bernardo M., Cione F., Capasso L., Coppola A., Rosa N. (2022). A formula to improve the reliability of optical axial length measurement in IOL power calculation. Sci. Rep..

[54] Kane J.X., Melles R.B. (2020). Intraocular lens formula comparison in axial hyperopia with a high-power intraocular lens of 30 or more diopters. J. Cataract Refract. Surg..

[55] Nemeth G., Modis L. (2021). Accuracy of the Hill-radial basis function method and the Barrett Universal II formula. Eur. J. Ophthalmol..

